# Serum leptin and adiponectin levels correlate with mast cell activation during exercise-induced bronchospasm in asthmatic children

**DOI:** 10.1186/1939-4551-8-S1-A26

**Published:** 2015-04-08

**Authors:** Jae-Won Oh, Ha-Baik Lee, Jae-Hyung Choi

**Affiliations:** 1 Hanyang University Kuri Hospital , South Korea

## Background

The aim of this study was to address the correlation between leptin, adiponectin and exercise induced bronchospasm (EIB) by measuring urinary metabolites of mast cell mediators such as 9α,11β-PGF_2_, LTE_4_.

## Methods

Seventy-two prepubertal children from the ages of 6 years to 10 years were recruited in the study. They comprised: asthmatic with EIB (n=24), asthmatic without EIB (n=21), and healthy controls (n=27). We measured exhaled nitric oxide (eNO) and serum eosinophilic cationic protein (ECP), leptin, adiponectin and cytokines. The urinary concentrations of LTE_4_ and 9α,11β-PGF_2_ were measured. The present study also performed pulmonary function tests: baseline, post-bronchodilator inhalation, methacholine inhalation and exercise. The area under the forced expiratory volume in one second (FEV_1_)-time curve quantified the severity of EIB over a 20-minute period after exercise (AUC_20_).

## Results

The post-exercise urinary excretion of 9α,11β-PGF_2_ in the asthmatics with EIB increased significantly compared with asthmatics without EIB. The post-exercise urinary excretion of LTE_4_ was not significantly difference between the two groups. The maximal decreases in % FEV_1_ after exercise were positively correlated with leptin levels and negatively with serum adiponectin levels in asthmatic children. Leptin presented positive associations correlated with post-exercise urinary excretion of 9α,11β-PGF_2,_ LTE_4_ and adiponectin presented negative associations correlated with post-exercise urinary excretion of LTE_4_

## Conclusions

Serum concentrations of the adipocyte-derived hormones leptin and adiponectin are correlated with EIB/BHR and urinary metabolites of mast cell mediators induced by exercise challenge in asthmatic children.

